# The Puzzle of Emotion Regulation: Development and Evaluation of the Tangram Emotion Coding Manual for Children

**DOI:** 10.3389/fpsyt.2019.00723

**Published:** 2019-10-11

**Authors:** Julie Hagstrøm, Katrine S. Spang, Bianca Munkebo Christiansen, Katrine Maigaard, Signe Vangkilde, Barbara Hoff Esbjørn, Jens Richardt Møllegaard Jepsen, Kerstin Jessica Plessen

**Affiliations:** ^1^Child and Adolescent Mental Health Centre, Mental Health Services, Capital Region of Denmark, Copenhagen, Denmark; ^2^Department of Clinical Medicine, Faculty of Health and Medical Sciences, University of Copenhagen, Copenhagen, Denmark; ^3^The Lundbeck Foundation Initiative for Integrative Psychiatric Research (iPSYCH), Aarhus, Denmark; ^4^Center for Anxiety, Department of Psychology, University of Copenhagen, Copenhagen, Denmark; ^5^Danish Research Centre for Magnetic Resonance, Centre for Functional and Diagnostic Imaging and Research, Copenhagen University Hospital, Hvidovre, Denmark; ^6^Center for Visual Cognition, Department of Psychology, University of Copenhagen, Copenhagen, Denmark; ^7^Center for Clinical Intervention and Neuropsychiatric Schizophrenia Research (CINS) and Center for Neuropsychiatric Schizophrenia Research (CNSR), Mental Health Centre Glostrup, University of Copenhagen, Copenhagen, Denmark; ^8^Division of Child and Adolescent Psychiatry, Department of Psychiatry, Lausanne University Hospital, Lausanne, Switzerland

**Keywords:** emotion regulation, development, interrater reliability, intrarater reliability, construct validity, convergent validity, discriminant validity, content validity

## Abstract

The ability to regulate one’s emotions is crucial to engaging successfully in social contexts. Difficulties in emotion regulation are seen in multiple psychiatric disorders, prompting an increased interest in the concept. Suitable methods for assessing emotion regulation, however, are lacking. In this study, we investigated the interrater and intrarater reliability, construct validity, and content validity of a new observational method for evaluating children’s emotion regulation abilities (a complex puzzle task) in a sample of 62 children without psychiatric disorders and 23 children with attention-deficit/hyperactivity disorder (ADHD) aged 7–12, using intra-class correlation coefficients for the reliability analyses and Spearman’s rank-order correlations for analyses of convergent and discriminant validity. A panel of experts examined the content validity of the test, and Mann–Whitney *U*-tests were used to investigate the ability of the test to differentiate the non-clinical group from the ADHD group. Results showed a high level of interrater and intrarater reliability of the test. There was mixed evidence for convergent and discriminant validity as expected due to the novelty and experimental nature of the test, making it difficult to compare with questionnaire-based measures. Content validity analysis was satisfactory, and the group comparison showed that the test differentiated the groups on the primary outcome measure. Overall, the measure demonstrated high feasibility and satisfactory psychometric properties. The generic nature of the test makes it suitable for use across psychiatric disorders and age groups with potential relevance in both research and clinical settings

## Introduction

Interest in the concept of emotion regulation (ER) has increased markedly over the years, making it one of the fastest-growing areas in the field of psychology (1). ER is an essential concept in psychological research and crucial for understanding the dimensions of mental illness (1–4), due to its pertinence on a continuum from normal development to severe psychopathology throughout life. Specifically, the ability to adequately regulate emotional responses is crucial for development and for the capacity to engage in social contexts (5). Also, disturbances in the development of self-regulatory control (including ER) most likely play a part in the development of a wide range of psychiatric disorders (6), and emotional dysregulation is involved in a great number of both externalizing and internalizing disorders, such as attention-deficit/hyperactivity disorder (ADHD) (7), anxiety, and depression (8). Finally, a key component in the growing interest in ER is its potential role in a dimensional approach to psychopathology (9, 10), exploring pathological processes in a transdiagnostic perspective to disentangle which impairments span multiple disorders and which are disorder specific (9). Despite the relevance and popularity of the concept, various definitions of both emotions and their regulatory processes exist in the vast literature. One widely used definition describes ER as the attempt to influence the experience and expression of one’s emotions through the activation of instinctive or explicit goals, which impacts the modification of an emotional response (1, 4, 11). Despite the growing interest in the field, research on the subject is sparse, possibly due to a lack of proper behavioral instruments of assessment (5). Several instruments for the assessment of ER exist, but these measures are either developed for younger children or adults or rely on indirect measures, such as parent-reported questionnaires. While self-report measures exist in several versions and remain the norm (12), this type of measure is far from ideal when examining ER (13), as self-report relies on the metacognitive abilities of the informant and is known to be affected by mood. Various neurobiological ways of investigating ER exist as well; however, more precise experimental measures are needed that allow for direct observation of the child’s behavior in a naturalistic setting to assess ER ability or screen for the lack of it.

We thus developed the Tangram Emotion Coding Manual (TEC-M) to contribute to the field with a clinical instrument to evaluate children’s ER profiles within the framework of parent–child interactions. The primary outcome of the test is the child’s overall ER ability, but other facets such as communication of emotions, specific regulatory strategies, and parental behavior are part of the instrument as well. The inspiration for this manual was found in the Tangram Construction Task Manual (Esbjørn et al., unpublished manuscript), which expands on previous methodology in the field of child anxiety (Tangram Task; 14, 15) and attachment relations (Co-Construction Task; 16). The current instrument, however, is based on the process model of emotion regulation (1, 11), which is a widely used framework for understanding and organizing ER. The process model centers on five sequential processes of ER, namely, “situation selection,” “situation modification,” “attentional deployment,” “cognitive change,” and “response modulation” (1, 3), which may range from adaptive to maladaptive depending on the specific individual, the context, and the emotions in question (11). Situation selection refers to choosing whether to engage in a given situation depending on the emotion that it is likely to elicit, whereas situation modification refers to directly altering the physical environment in a given situation. Attentional deployment captures the possibility of directing attention towards or away from specific stimuli to influence one’s emotions. Cognitive change refers to how individuals appraise a situation to alter its emotional significance. Finally, response modulation occurs late in the emotion-generative process and captures the individual’s experiential, physiological, or behavioral responses to the situation eliciting a given emotion (11). This model shaped the development of the coding manual by elucidating specific aspects of the regulatory process that may occur during regulation of emotions, thus providing a framework for items to be included. This allowed for a combined theory- and data-driven process of item generation, in which we included items representing observable aspects of each process.

As part of the validation of the instrument, we investigated the construct validity (comprising convergent and discriminant validity) of the TEC-M, which reflects the degree to which an instrument measures what it purports to measure (17). Convergent validity deals with the association between the test under investigation and a theoretically related variable (18), whereas discriminant validity is supported by the finding of smaller or non-existing associations with scores from instruments that are not directly theoretically related (18), thus supporting the uniqueness of a new measure (19). Several studies have suggested a link between executive functions and ER. From a neural perspective, both tasks require activation of the frontal lobes, and one hypothesis states that executive planning strategies interact with the individual’s underlying affective characteristics to facilitate successful ER (20). Zelazo and Cunningham (21) suggested that the conscious and voluntary form of ER simply is an executive task making the two constructs isomorphic. Based on the association between executive functions and ER, we hypothesized that the Emotional Control Scale (EC) from the Behavior Rating Inventory of Executive Function (BRIEF) (22) would correlate well with the overall ER outcome of the instrument. Additionally, we chose the Deficient Emotional Self-Regulation (DESR) profile (23, 24) from the Child Behavior Checklist (CBCL) (25) and expected a similar correlation, as this scale reflects elevated scores on a number of symptoms assumed to reflect general dysregulation of emotions. We chose IQ for the analysis of discriminant validity. Based on the nature of the test, we expected some correlation between ER and the intellectual abilities of the child. Previous studies have demonstrated associations between children’s parent-reported ER skills and later academic success (26), as well as ER abilities and kindergarten achievement (27). One study with adults, however, reported only weak correlations between IQ and emotional intelligence scores (28). We thus expected some overlap in IQ and ER as measured with the TEC-M but assumed this association weaker than that between ER and theoretically related questionnaire measures.

The main objectives of the present study were to describe the development of a new clinical instrument that accommodates the need for observational, clinician-rated evaluation of children’s ER profiles and to assess the interrater reliability (IRR) and intrarater reliability of the instrument, as well as its convergent, discriminant, and content validity. Finally, we examined the instrument’s ability to differentiate ER profiles in a group of children with ADHD from a typically developing control group. Children with ADHD are known to present with dysregulated behavior (29), and we were interested in whether the test would be sensitive to this. We hypothesized that the group of children with ADHD would achieve lower scores of overall emotion regulation ability.

## Methods

### Participants

Participants were 85 children aged 7–12 (*M* = 9.42, *SD* = 1.36). The vast majority lived in the Capital Region (*N* = 77) with the remainder living in other regions of Denmark. Of the 85 included children, 62 were healthy controls, and 23 children had a diagnosis of ADHD, eight of which of the predominantly inattentive type (**Table 1**). Five children with ADHD additionally met diagnostic criteria for oppositional-defiant disorder (ODD). The gender distribution showed a predominance of boys due to the fact that the majority of control participants were matched to a population of children with neuropsychiatric disorders presenting with a natural preponderance of boys. All participants underwent a thorough clinical assessment with the Kiddie-Schedule for Affective Disorders and Schizophrenia, Present and Lifetime Version (K-SADS-PL) (30). Control participants were excluded if they met Diagnostic and Statistical Manual of Mental Disorders, fourth edition (DSM-IV) (31) criteria for any lifetime psychiatric diagnosis with the exception of enuresis and encopresis. Children with ADHD were excluded if a comorbid autism spectrum disorder or psychotic disorder was present. All participants were Caucasian except for one participant of South Asian descent, and no participants were taking or had previously been taking psychotropic medications.

**Table 1:** Participant characteristics.

Characteristic	ADHD (*n* = 23)	Controls (*n* = 62)	Test of group differences
Male, *N* (%)	15 (65.2)	49 (79.0)	*p* = .190^a^
Mean age (*SD*)	9.59 (1.25)	9.36 (1.40)	*p* = .488^b^
Comorbid disorders^c^, *N* (%)	6 (26.1)	0 (0)	

ADHD, attention-deficit/hyperactivity disorder.

^a^Chi-square test of homogeneity. ^b^t(83) = 0.697. ^c^Four children with ADHD additionally met criteria for oppositional defiant disorder (ODD), one participant had both ODD and separation anxiety disorder, and one participant met criteria for generalized anxiety disorder, separation anxiety disorder, and transient tic disorder in addition to ADHD.

Participants were drawn from two larger studies carried out in the Mental Health Services, Capital Region of Denmark. Twenty-one children with ADHD and all children aged 8–12 (*N* = 70) were part of a study investigating ER in children with neuropsychiatric disorders, whereas the remaining 13 control participants and two participants with ADHD were part of a control group of 7-year-old children from a high-risk study (32). Children with ADHD were recruited from the local outpatient clinic when referred for diagnostic assessment and were tested before starting any treatment (medical or therapeutic). Two children with ADHD were initially recruited as controls but met criteria for a diagnosis during the assessment. We chose to include participants from two separate studies to increase the age span. Participants were recruited from 2013 to 2016. All healthy controls from the two studies were randomly selected and recruited *via* the Danish Civil Registration System (33) to reduce selection bias.

### Measures

#### The Tangram Construction Task

The Tangram Construction Task is a 5-min test that is administered with the child and one caregiver present. The test has been carried out successfully with children from the age of 7 to 12 but could potentially be used in both younger and older age groups. During the 5 min, the child is instructed to solve as many puzzles as possible from a total of six puzzles. The parent is instructed to help only if it is “truly necessary” and is given the solution booklet to aid the process without being allowed to show the solutions to the child. The test administrator leaves the room after having instructed the child and parent. The 5-min test is videotaped for subsequent coding. Debriefing at the end of the test emphasizes for the child that the task is extremely difficult even for adults and that the objective was to see how children carry on with their work even when it poses great challenges. The brevity of the test makes it suitable for research and in clinical assessment when mapping children’s abilities to regulate emotions. Furthermore, the presence of a parent increases the ecological validity by mimicking a real-life situation where the child is alone with a parent instead of facing an unfamiliar test administrator.

The present manual (available upon request) comprises eight items for parental behavior (intrusiveness, control, avoidance, verbal reappraisal, support sensitivity, positive expressions, negative expressions, and tension), nine items for child behavior [control, avoidance/resignation, narration, verbal reappraisal, reassurance-seeking behavior, aggression, positive expressions, negative expressions, and the overall ER scale (EmReg)], and one item on the parent–child dyad (emotional warmth). Each regulatory process of the process model of emotion regulation is represented by at least one item that is placed accordingly on the scoring sheet, although in theory the boundaries between the five processes can be fluid and some items may be applicable to multiple processes. Although several facets of parental behavior are scored, the primary focus of the test is child behavior. The purpose of the parent items is to allow for the possibility of studying the quality of interaction (represented by the item “emotional warmth”) and to have access to parental measures of potential interest in clinical settings such as negative attitudes or level of support. Also, parental behavior is taken into account when assessing the child’s overall ER ability by weighing child behavior against the displayed parental support or lack thereof. Three additional items for child behavior (situation rejection, tension, and incongruent positive affect) appear in the present analyses but were ultimately removed as a result of the work presented in this article. All items except for the EmReg score are scored on a 4-point Likert scale on frequency (never, rarely, sometimes, and often) and on a 3-point Likert scale on intensity (mild, moderate, and marked). The manual offers a system for coding frequency as well as descriptions of the types of behavior that warrant specific intensity scores. For example, for the item of reassurance seeking, non-verbal reassurance seeking (such as glancing up at the parent) will generate a score of 1; indirect, verbalized reassurance seeking (such as stating that the task is difficult without directing it at the parent) will generate a score of 2; and direct, verbalized reassurance seeking (requests for help) generates a score of 3. The EmReg score is an overall measure of the child’s perceived ER skills when taking into account all factors, such as parental influence and perceived difficulties, and it is scored on a 5-point scale with 1 representing very poor ER skills and 5 representing excellent ER skills. The remaining items cover aspects of ER such as the use of specific strategies, but also more simple emotional behavior such as emotion communication as well as behavior comprising a more executive component. This way of coding allows for a quantification of the child’s abilities and a descriptive insight into the personal ER style. The inclusion of the EmReg score allows for measuring changes over time, for example, before and after therapy.

#### BRIEF (Parent Form)—Emotional Control Scale

The BRIEF is a parent-completed rating scale consisting of 86 questions that assess executive function in children and adolescents aged 5–18 (22). Parents are asked to rate their child’s problematic behaviors during the past 6 months. The EC scale is one of eight clinical scales in the BRIEF and reflects the influence of executive function on ER.

#### CBCL—”Deficient Emotional Self-Regulation Profile”

Parents completed the CBCL for ages 6–18, which is a questionnaire identifying various problem behaviors in children (25). Parents were asked to rate their child’s behavior during the past 6 months. The DESR profile from the CBCL combines the scores of three symptom scales (aggressive behavior, anxious/depressed, and attention problems) and has been found to reflect difficulties in regulation of emotions, as well as being associated with high rates of disruptive behavior (23, 24).

#### Wechsler Intelligence Scale for Children

The fourth edition of the Wechsler Intelligence Scale for Children (WISC-IV) is an intelligence test for children aged 6–16, which generates a full-scale IQ and a number of index scores (34). In the present study, we used the full-scale IQ along with the Verbal Comprehension Index (VCI) and the Perceptual Reasoning Index (PRI). The former measures aspects of intelligence reflected in verbal concept formation, and the latter is a non-verbal measure of facets of intelligence reflected in fluid reasoning, spatial processing, and visual–motor integration.

### Procedures

The study was approved by the Regional Committee on Health Research Ethics (journal number H-2-2013-085) and the Danish Data Protection Agency (journal number 2017-58-0015). Written informed consent was obtained from parents of all participants in accordance with the Declaration of Helsinki. All subjects participated in the test as part of a large test battery. Psychologists, medical doctors, or a research nurse administered the test and gave instructions verbatim in order to decrease instruction bias. Two trained raters (JH and KS) conducted the coding of the videos during a period of 3 months.

### Data Analyses

Statistical analyses were performed using SPSS version 22.0. The datasets for the analysis in this manuscript are available upon request, without reservations, to all researchers.

We used the intra-class correlation coefficient (ICC) for the analysis of IRR. This is one of the most frequently used statistics for reporting IRR for ordinal variables (35). Two trained raters (JH and KS) coded a subset of 30 participants for the IRR analysis. For the intrarater reliability analysis, each rater coded 10 videos twice with approximately 3 months in between. We applied a two-way mixed model testing for absolute agreement with average-measures ICCs. Reliability coefficient values were interpreted based on the guidelines presented in Cichetti (36).

We conducted Spearman (37) rank-order correlations, as EmReg is both an ordinal variable and presented with a non-normal distribution. We tested associations between the EmReg score and the EC Scale, the DESR profile, total IQ, VCI, and PRI. For the comparison with IQ, only the 70 participants drawn from the study with children with neuropsychiatric disorders were included, as the 15 children from the high-risk study were not tested with the WISC-IV. Values of ±0.1 represent a small effect, ± 0.3 is a medium effect, and ±0.5 is a large effect (38). We chose only the EmReg score for comparison as this representation of overall ER ability is the primary outcome of the TEC-M.

The concept of content validity centers on the theoretical construct that is being measured and whether a test is able to capture all aspects of that construct (39). Lawshe (40) proposed a method of testing content validity, which makes use of a panel of experts in the field who evaluate individual items of a test as to whether they reflect the theoretical domain in a satisfactory manner. In this method, the panel rates each item as either “essential,” “useful, but not essential,” or “not necessary.” From these ratings, it is possible to calculate a content validity ratio (CVR), which is held against a critical value depending on the number of panelists. In the present study, eight panelists at the doctoral and master’s levels rated the items, requiring a CVR of 0.75. Any item perceived essential by more than half of the panelists is thought to hold some degree of content validity, and the more who perceive it to be essential, the greater its content validity (40).

We examined the ability to differentiate the ADHD group from the control group for all items. We used Mann–Whitney *U* tests (41) to investigate group differences, as items were scored on an ordinal scale, and calculated effect sizes for significant results. Differences in age and gender between the control group and the ADHD group were assessed with an independent-samples *t*-test and the chi-square test of homogeneity, respectively.

## Results

### IRR and Intrarater Reliability

ICCs showed excellent agreement for 11 items, good agreement for 13 items, fair agreement for 6 items, and poor agreement for 4 items (**Table 2**). The intrarater reliability analyses showed excellent agreement for respectively 23 and 24 items, good agreement for 5 items, fair agreement for respectively 3 and 4 items, and poor agreement for 1 item with some variation in items between raters. For items with zero or low variance, an ICC could not be calculated. Overall, the ICCs reflected a high level of interrater and intrarater agreement.

**Table 2:** Interrater and intrarater reliability.

Item (F = frequency, I = intensity)		Distribution of scores (%)				Interrater reliability		Intrarater reliability (rater 1)		Intrarater reliability (rater 2)	
		0	1	2	3	ICC	95% CI	ICC	95% CI	ICC	95% CI
Parent items	Intrusiveness F	30	33	15	22	**0.71**	[0.53, 0.89]	**0.88**	[0.51, 0.97]	**0.84**	[0.41, 0.96]
	Intrusiveness I	30	43	18	8	**0.76**	[0.31, 0.90]	**0.83**	[0.33, 0.96]	**0.91**	[0.62, 0.98]
	Avoidance F	73	25	2	0	**0.79**	[0.57, 0.90]	**1.00**	N/A	**0.90**	[0.62, 0.97]
	Avoidance I	73	20	7	0	**0.78**	[0.54, 0.89]	**1.00**	N/A	**0.85**	[0.29, 0.96]
	Control F	0	5	28	67	**0.63**	[0.22, 0.83]	**0.63**	[−0.43, 0.91]	**0.82**	[0.29, 0.95]
	Control I	0	13	67	20	**0.76**	[0.50, 0.88]	0.52	[−0.63, 0.87]	**0.71**	[−0.02, 0.93]
	Verbal reappraisal F	73	18	8	0	**0.89**	[0.76, 0.95]	**0.94**	[0.77, 0.98]	**1.00**	N/A
	Verbal reappraisal I	73	18	7	2	**0.73**	[0.43, 0.87]	**0.89**	[0.59, 0.97]	**0.91**	[0.68, 0.98]
	Negative expressions F	50	40	10	0	0.50	[−0.05, 0.76]	**0.74**	[0.07, 0.93]	**0.83**	[0.33, 0.96]
	Negative expressions I	50	40	8	2	0.48	[−0.10, 0.75]	**0.78**	[0.20, 0.94]	**0.76**	[−0.07, 0.94]
	Positive expressions F	3	28	35	33	**0.64**	[0.26, 0.83]	**0.83**	[0.29, 0.96]	**0.96**	[0.86, 0.99]
	Positive expressions I	3	50	43	3	0.58	[0.12, 0.80]	**0.94**	[0.77, 0.98]	0.54	[−1.01, 0.89]
	Tension F	93	3	2	2	0.55	[0.03, 0.78]	**1.00**	N/A	**1.00**	N/A
	Tension I	93	2	5	0	**0.75**	[0.47, 0.88]	**1.00**	N/A	**1.00**	N/A
	Support sensitivity F	2	12	30	57	**0.75**	[0.39, 0.89]	0.57	[−1.02, 0.90]	**0.65**	[−0.26, 0.91]
	Support sensitivity I	2	25	62	12	0.22	[−0.61, 0.62]	**0.69**	[−0.08, 0.92]	0.53	[−1.27, 0.89]
Child items	Situation rejection F	100	0	0	0	ZV	N/A	ZV	N/A	ZV	N/A
	Situation rejection I	100	0	0	0	ZV	N/A	ZV	N/A	ZV	N/A
	Control F	0	7	43	50	**0.66**	[0.28, 0.83]	**0.89**	[0.52, 0.97]	**0.74**	[0.00, 0.93]
	Control I	0	32	63	5	0.51	[−0.02, 0.76]	0.37	[−1.89, 0.85]	0.40	[−2.03, 0.86]
	Avoidance/resignation F	38	28	28	5	**0.61**	[0.16, 0.82]	**0.95**	[0.80, 0.99]	**0.66**	[−0.49, 0.92]
	Avoidance/resignation I	38	27	35	0	**0.70**	[0.33, 0.86]	**0.91**	[0.66, 0.98]	0.36	[−1.03, 0.83]
	Narration F	52	32	17	0	**0.64**	[0.25, 0.82]	**0.76**	[0.04, 0.94]	**0.80**	[0.27, 0.95]
	Narration I	52	13	35	0	**0.67**	[0.31, 0.84]	**0.86**	[0.42, 0.96]	**0.80**	[0.27, 0.95]
	Verbal reappraisal F	70	25	3	2	**0.60**	[0.17, 0.81]	**1.00**	N/A	**0.84**	[0.33, 0.96]
	Verbal reappraisal I	70	17	13	0	**0.71**	[0.39, 0.86]	**0.97**	[0.88, 0.99]	**0.85**	[0.43, 0.96]
	Reassurance-seeking F	10	27	43	20	**0.83**	[0.65, 0.92]	**0.83**	[0.32, 0.96]	**0.90**	[0.57, 0.98]
	Reassurance-seeking I	10	30	55	5	0.40	[−0.28, 0.72]	**0.69**	[−0.18, 0.92]	**0.68**	[−0.38, 0.92]
	Tension F	98	2	0	0	ZV	N/A	ZV	N/A	ZV	N/A
	Tension I	98	2	0	0	ZV	N/A	ZV	N/A	ZV	N/A
	Aggression F	96	3	0	0	−0.07	[−1.35, 0.50]	ZV	N/A	**0.78**	[0.17, 0.94]
	Aggression I	97	3	0	0	−0.07	[−1.35, 0.50]	ZV	N/A	**0.78**	[0.17, 0.94]
	Negative expressions F	3	33	40	23	**0.72**	[0.41, 0.87]	0.47	[−0.77, 0.86]	**0.93**	[0.73, 0.98]
	Negative expressions I	3	42	48	7	**0.62**	[0.22, 0.82]	**0.73**	[−0.14, 0.93]	**1.00**	N/A
	Positive expressions F	7	28	42	23	**0.78**	[0.53, 0.90]	**0.78**	[0.08, 0.95]	**0.80**	[0.15, 0.95]
	Positive expressions I	7	45	45	3	**0.76**	[0.49, 0.89]	**1.00**	N/A	**0.83**	[0.37, 0.96]
	IPA F	98	2	0	0	ZV	N/A	ZV	N/A	ZV	N/A
	IPA I	98	2	0	0	ZV	N/A	ZV	N/A	ZV	N/A
Emotional warmth		0	3	20	77	0.33	[−0.23, 0.66]	**1.00**	N/A	0.54	[−1.01, 0.89]
EmReg [score (*n*)]		1 (1), 2 (11), 3 (31), 4 (32), 5 (10)				**0.78**	[0.53, 0.89]	**0.81**	[0.21, 0.95]	**0.79**	[0.10, 0.95]

CI, confidence interval; ICC, intra-class correlation coefficient; ZV, zero variance; N/A, not applicable. IPA, incongruent positive affect; EmReg, overall emotion regulation scale. All ICC values of good or excellent agreement are marked in bold (36).

### Convergent and Discriminant Validity

For convergent validity, we found a weak and only trend-level, inverse correlation between EC and the EmReg score, *r* *_s_*(78) = −.20, *p* = .079 (**Table 3**). Regarding the DESR profile compared to EmReg, we found a significant, close to medium, inverse correlation, *r* *_s_*(73) = −.28, *p* = .016. For the comparison with EC and DESR, data were missing from 5 and 10 participants, respectively, whose parents had not filled out the questionnaires. We found a medium, positive correlation between total IQ and EmReg score, *r* *_s_*(68) = .30, *p* = .011, for the analysis of discriminant validity. Weak positive correlations were found between EmReg and the VCI, *r* *_s_*(68) = .27, *p* = .026, and the PRI, *r* *_s_*(68) = .28, *p* = .018.

**Table 3:** Spearman’s rank-order correlations.

		EC	DESR	Total IQ	VCI	PRI
EmReg	Correlation	−.20	−.28	.30	.27	.28
	*p* value	.079	.016	.011	.026	.018

EmReg, overall emotion regulation scale; EC, Emotional Control Scale; DESR, Deficient Emotional Self-Regulation profile; VCI, Verbal Comprehension Index; PRI, Perceptual Reasoning Index.

### Content Validity

For the content validity analysis (**Table 4**), seven items out of a total of 21 reached the critical value of 0.75 (situation rejection, control, narration, verbal reappraisal, reassurance-seeking behavior, aggression, and the EmReg score). At least half the panelists perceived eighteen items in total as essential, suggesting broad basis for content validity. For three items, more than half the panelists rated ‘Useful, but not essential’, whereas only one item was perceived as ‘Not necessary’ by only one panel member, and this item was ultimately removed.

**Table 4:** Content validity.

Item	Rating count^*^	CVR
Parental intrusiveness	5; 3; 0	0.25
Parental avoidance	5; 3; 0	0.25
Parental control	6; 2; 0	0.5
Parental verbal reappraisal	6; 2; 0	0.5
Parental negative expressions	4; 4; 0	0
Parental positive expressions	4; 4; 0	0
Parental tension	3; 5; 0	−0.25
Parental support sensitivity	6; 2; 0	0.5
Child situation rejection	8; 0; 0	1
Child control	8; 0; 0	1
Child avoidance/resignation	6; 2; 0	0.5
Child narration	7; 1; 0	0.75
Child verbal reappraisal	8; 0; 0	1
Child reassurance-seeking behavior	8; 0; 0	1
Child tension	5; 3; 0	0.25
Child aggression	8; 0; 0	1
Child negative expressions	6; 2; 0	0.5
Child positive expressions	6; 2; 0	0.5
Child incongruent positive affect	3; 4; 1	−0.25
Emotional warmth	3; 5; 0	−0.25
EmReg	7; 1; 0	0.75

*”Essential”; “Useful, but not essential”; “Not necessary”. CVR, content validity ratio; EmReg, overall emotion regulation scale.

### Sensitivity Between Groups

There was homogeneity of variances for age as assessed by Levene’s test for equality of variances (*p* = .497). The two groups did not differ on age and sex distributions (**Table 1**). Mann–Whitney *U* tests were run to determine which items could differentiate the ADHD group from the control group. Distributions of item scores for children with ADHD and those in the control group were similar when assessed by visual inspection. For all significant scores, both the median and the mean rank are reported, as the Likert-scale format of the ratings can lead to identical medians for some items, even when there is a difference between groups. Item scores differed significantly between the groups on seven out of a total of 40 items: Item score on frequency of parental tension was significantly higher in the ADHD group (*Mdn* = 0.00, mean rank = 47.15) than in the control group (*Mdn* = 0.00, mean rank = 41.46), *U* = 808.5, *z* = 1.982, *p* = .047, *r* = 0.21. Item score on frequency of child control was significantly higher in the control group (*Mdn* = 3.00, mean rank = 46.60) than in the ADHD group (*Mdn* = 2.00, mean rank = 33.30), *U* = 409, *z* = −2.566, *p* = .010, *r* = −.28. For frequency of child narration, the control group (*Mdn* = 1.00, mean rank = 47.21) scored significantly higher than the ADHD group (*Mdn* = 0.00, mean rank = 31.65), *U* = 452, *z* = −2.885, *p* = .004, *r* = −0.31, and the same was true for intensity of child narration where the control group (*Mdn* = 1.00, mean rank = 46.44) scored significantly higher than the ADHD group (*Mdn* = 0.00, mean rank = 33.72), *U* = 499.5, *z* = −2.366, *p* = .018, *r* = −.26. For frequency and intensity of child aggression (identical results), the ADHD group scored higher (*Mdn* = 0.00, mean rank = 45.70) than the control group (*Mdn* = 0.00, mean rank = 42.00), *U* = 775, *z* = 2.366, *p* = .019, *r* = .26. Finally, children from the control group achieved significantly higher scores on the EmReg (*Mdn* = 4.00, mean rank = 47.81) than the ADHD group (*Mdn* = 3.00, mean rank = 30.02), *U* = 414.5, *z* = −3.122, *p* = .002, *r* = −.34.

## Discussion

The purpose of this study was two-fold. Firstly, we described the theoretical background and development of the TEC-M; a novel coding manual for an experimental test of ER in children that allows for direct, observational measures of overall ER ability as well as a number of ER characteristics and quality of the parent-child interaction. Secondly, we examined the manual on a number of parameters, such as reliability, validity, and sensitivity to group differences.

Analyses showed good or excellent interrater agreement for the majority of items pointing to a substantial robustness of the items and indicating that training and consulting the manual can produce reliable ratings on most items between two professional groups (a psychologist and a medical doctor). The items reaching only poor or fair agreement point to a need for clarification in the manual and further standardized assessment of the respective scores. For the five items with poor reliability, we propose different explanations for these results. Regarding the items of aggression, this type of behavior was only scored positive for one child, rendering the analysis particularly sensitive to deviations. For the remaining items, anchor points for the various scores had not initially been described sufficiently, thus decreasing the objectivity of the scores. Results from the intrarater reliability analysis showed that the two raters succeeded in similar or identical ratings at two points in time for the vast majority of items. For the few items that reached poor or fair agreement (only one item per rater showed poor agreement), the majority were intensity scores, suggesting a need for further clarification of the different levels of intensity of a specific behavior. Although reliability ratings were satisfactory, it should be emphasized that thorough training in the coding procedures is central to decreasing bias in clinician ratings by creating a common framework for coding. Additionally, regular co-ratings with experienced coders would be preferable to strengthen the accuracy of clinicians’ ratings. Finally, an important point for future studies would be to assess IRR between more than two raters. Three items showed zero variance in the distribution of scores (child situation rejection, child tension, and incongruent positive affect), which led to the decision of removing them from the manual completely as the rarely displayed behavior pertaining to these items was well-represented by other items.

There are no clear guidelines as to which scores might serve as cut-off for establishing construct validity when interpreting correlations. Cichetti (36) accurately points out that the ideal correlation value depends on what the new test purports to measure in relation to an existing one: “We know for sure that we would hope for a correlation of neither 1.00 nor 0. In the first case, the new test could be considered a veritable clone of the one with which it is being compared. In the second case, the construct validity of the very concept being measured would be called into question” (36, p. 5). Both correlations for convergent validity (EmReg compared to EC and DESR) were weak; however, the comparison with DESR was close to medium and significant. Due to the explorative nature of these tests we did not correct for multiple comparisons, but it would be interesting to see if future studies would be able to replicate these findings. Although traditionally correlations testing for convergent validity are higher, it is not surprising that these correlations were not. First of all, the questionnaires used for comparison were parent-reports of child behavior assessed over a period of 6 months. This type of assessment, though theoretically intended to measure ER, is in clear contrast to the outcomes of the TEC-M which aims at direct and instantaneous observation of ER in a concrete setting within a limited timeframe. The purpose of developing the TEC-M was to create an instrument that was able to capture aspects of ER that previous studies had not been able to, due to a lack of tests or relying on parent- or self-report measures. Future studies of the validity of the TEC-M would benefit from more similar measures of comparison; however, the current lack of experimental tests in the area complicates the possibility for investigating convergent validity further. With regard to the examination of discriminant validity, the correlation between IQ (full scale IQ, VCI, and PRI) was in the medium range, suggesting some association between performance on the test (ER ability) and general intellectual abilities as shown in previous studies. We expected that the experienced level of frustration would to some degree depend on how difficult the child would find the task, thus explaining the association with IQ. Interestingly, the correlations between the EmReg score and VCI and PRI, respectively, were almost identical. We expected to find a slightly higher correlation between EmReg and PRI as the tasks covered in this index are somewhat similar to the Tangram Construction Task. The TEC-M, however, also includes aspects related to verbal abilities, such as narration and verbal reappraisal. The fact that verbal comprehension and perceptual reasoning correlated equally well with EmReg supports the role of the EmReg score not as a measure of construction skills, but as a measure of ER ability. A limitation associated with the use of IQ is its holistic nature, making it very likely to produce correlation coefficients in the present range with most psychological tests. Although it is highly relevant to establish that level of frustration and the ability to regulate this frustration are not directly dependent on how capable the child is in solving the task, a more specific test within the domain of executive functions or memory, for example, could have been superior in establishing discriminant validity.

For the examination of content validity, more than half the panelists rated the majority of items “essential,” displaying a satisfying degree of content validity, although the critical CVR was only reached for seven items. All items except for one were rated either “essential” or “useful, but not essential” supporting the relevance of the selected items for the reflection of the theoretical construct of ER. The panelists were asked to rate the relevance of each item in regard to its ability to adequately cover ER, leading to some panelists finding the parent items to be only useful, but not essential as they were not direct measures of the child’s abilities. The same was the case for the item covering “emotional warmth,” which is a shared measure for the child and the parent and as such does not capture the specific ER abilities of the child. Still, we find the inclusion of parent items relevant for elucidating the bigger picture of children’s ER characteristics and the influence of parental behavior on these behaviors, an association that has been established in research previously (5).

We examined the test’s ability to differentiate children with ADHD from the control children in an explorative manner and found significant differences in scores between the groups on seven items with effect sizes in the medium range. These findings corresponded well with existing knowledge in the field of ADHD pointing to reduced ER abilities, an increased occurrence of aggressive behavior, as well as deficits in executive functioning (42). Firstly, the ADHD group scored significantly lower on the EmReg score, which is a measure of overall ER ability. Secondly, the ADHD group scored significantly higher on both frequency and intensity of aggression. This finding, however, must be interpreted with caution due to the low number of participants scoring higher than zero on these items. Frequency of parental tension was significantly higher in the ADHD group than in the control group, a finding that is in line with research showing a strong association between parenting styles and ADHD symptom severity (43). For the control group, scores were significantly higher on frequency of control, as well as frequency and intensity of narration. An explanation for this might be that both the autonomy of taking control of the task and the employment of narration to facilitate task solving rest on higher executive functions which may be impaired in children with ADHD. Due to the explorative nature of these analyses, with the exception of the hypothesis for the EmReg score, we did not control for multiple comparisons. The significant group difference on the EmReg score does, however, remain significant when applying the Benjamini–Hochberg false discovery rate method.

The TEC-M is not intended as a diagnostic test, but as a transdiagnostic tool to characterize children’s individual ER abilities through systematic rating. Although in the present study the test has only been applied to children with ADHD, the TEC-M is generic and can be used across diagnostic groups with a dimensional approach to dysregulation of emotions. The frustration element of the puzzle is necessary to facilitate the elicitation of codable behavior, but this premise also limits the degree to which one can make statements regarding general behavior. Interpreting the displayed behavior as part of assessment in a child psychiatric setting must therefore include a consideration of the limited context of the task and must always be viewed in connection with the complete assessment. In future studies, we plan to expand the contexts of assessment by adding test scenarios with the child being in the room with a test administrator, as well as further increasing the ecological validity by administering the test in the child’s home. The test could also easily be administered with both parents as well as a teacher to allow for a multifaceted examination of ER. In a clinical context, the test may serve as an evaluation before and after therapy or as a measure to assess the need for psychoeducation in the family. Additionally, using instructive examples from the videos to illustrate maladaptive and adaptive behaviors on behalf of both the parent and the child could prove useful in psychoeducation and therapy. Finally, the exploration of potential profiles of ER corresponding to various disorders could help accommodate a dimensional approach to child psychopathology and an individualized approach to treatment.

## Limitations

There are a number of limitations to this study, such as a preponderance of boys and a limited age span in the sample. However, due to its feasibility, the test could easily be used in both younger and older age groups and a general expansion of the participant sample in regard to gender, age, and clinical grouping represents an important direction for future research. The small sample size, particularly for the ADHD group, represents another limitation to this study, which could have affected the power of our analyses to detect significant differences between the groups. Moreover, future measures for convergent and discriminant validity should be chosen carefully beforehand to assure these correlations in the full sample of participants. One major limitation of the study was the lack of systematic blinding. As the raters had administrated the test themselves to some of the participants, it was impossible to achieve total blinding. Many of the participants, however, had been instructed by others than the raters and in the case of a rater having instructed a particular participant, several months would have passed between contact with the patient and the actual rating.

Five of the co-authors in the present study were simultaneously part of the expert panel for the examination of content validity. The main purpose of the rating, however, was not to reach a high CVR, but rather to evaluate the instrument in an unbiased manner as part of its development. Additionally, the panelists were kept in the dark as to how their ratings would translate into an assessment of content validity.

A final limitation to be discussed is the inherent challenge in measuring ER on a behavioral level, as the regulation itself will often be invisible to the rater. The TEC-M is designed to code the actual behavior exhibited, but it cannot declare the degree of regulation or the intensity of the emotion that is being regulated. The use of explicit strategies such as verbal reappraisal or narration is directly codable, whereas the implicit or nonverbal regulation will always be an approximation. The key goal of the EmReg score is to function as such an approximation by taking into account all information gathered from the TEC-M.

## Conclusion

The findings of the present study support the use of the TEC-M, a coding manual for an observational test of ER in children, by demonstrating satisfactory psychometric properties, although future studies are needed to examine construct validity further. This contribution is relevant as dysregulation of emotions is evident in numerous mental disorders and a further understanding of these processes might help improve treatment options. The TEC-M differs from existing measures in regard to its ecological validity and systematic observation of the child and its generic methodology will hopefully be able to contribute to a transdiagnostic approach to psychopathology focusing on a fundamental dimensionality in psychiatric disorders.

## Ethics Statement

The study that this research was part of was approved by the Regional Committee on Health Research Ethics. Written informed consent was obtained from parents of all participants, as participants were aged 7-12, in accordance with the Declaration of Helsinki. Special care was taken to ensure that the children felt safe and comfortable, and if any child wished to discontinue their participation in the study, the child would be excluded even with consent from the parents. As the purpose of the task in question was to cause some amount of frustration in the child (with a caregiver present), we followed up with immediate thorough debriefing with an explanation of the nature of the task to the child and parent.

## Author Contributions

JH collected the data, developed the instrument, and wrote the manuscript with input from all authors. KS co-developed the instrument and both KS and KM contributed to the collection of data. BC helped drafting a part of the text, and BE developed the original instrument on which this work was based. JJ and KP supervised the development of the instrument and contributed to the interpretation of the results, and SV and KP were involved in designing the study. All co-authors critically revised the article for intellectual content and approved the final version.

## Funding

This research was supported by a PhD scholarship from the Mental Health Services, Capital Region of Denmark.

## Conflict of Interest

The authors declare that the research was conducted in the absence of any commercial or financial relationships that could be construed as a potential conflict of interest.
